# Supplementary material for: Generation of Angiotensin-Converting Enzyme 2/Transmembrane Protease Serine 2-Double-Positive Human Induced Pluripotent Stem Cell-Derived Spheroids for Anti-Severe Acute Respiratory Syndrome Coronavirus 2 Drug Evaluation
Source: Microbiol Spectr. 2022 Oct 31;10(6):e03490-22. doi: 10.1128/spectrum.03490-22 (PMC9769601; doi:10.1128/spectrum.03490-22)
Supplement: Supplemental file 1 — Supplemental material. Download spectrum.03490-22-s0001.pdf, PDF file, 3.4 MB [file spectrum.03490-22-s0001.pdf]

Supplementary information

Generation of ACE2/TMPRSS2-double-positive-hiPSC-derived Spheroids for Anti-SARS-CoV-2 drug evaluation

Nobuyo Higashi-Kuwaya[a], Shigeharu G. Yabe[b], Satsuki Fukuda[b], Junko Nishida[b], Miwa Tamura-Nakano[c], Shin-ichiro Hattori[a], Hitoshi Okochi[b], Hiroaki Mitsuya[a]

[a]*Department of Refractory Viral diseases, National Center for Global Health and Medicine Research Institute, Shinjuku-ku, Tokyo 162-8655, Japan,*

[b]*Department of Regenerative Medicine, Research Institute, National Center for Global Health and Medicine, 1-21-1 Toyama, Shinjuku-ku, Tokyo, 162-8655, Japan*

[c]*Communal Laboratory, Research Institute, National Center for Global Health and Medicine, Tokyo 162-8655, Japan.*

[d]*Experimental Retrovirology Section, National Cancer Institute, NIH, Bethesda, MD, USA*

Ethical approval for this study was obtained from the institutional ethics committee.

Culture of undifferentiated hiPSCs

The TkDN4-M (4M) hiPSCs line was a kind gift from Dr. M Ohtsu at The Institute of Medical Science, The University of Tokyo. 4M was cultured on the mitomycin C (MMC; FUJIFILM Wako Pure Chemical Corporation, Osaka, Japan)-treated SNL feeder cells (ECACC, Salisbury, UK) in hiPSCs medium (DMEM/Ham`s F12 (FUJIFILM Wako) supplemented with 20% Knockout Serum Replacement (KSR; GIBCO BRL, Palo Alto, CA, USA), 1x MEM nonessential amino acids (FUJIFILM Wako), 0.5x penicillin streptomycin (PS; FUJIFILM Wako) , 55µM 2-melcaptoethanol (2ME; Gibco) and 7.5µg/ml basic fibroblast growth factor (FGF2; Peprotech, Rocky Hill, NJ, USA). For passage, 4M colonies were detached from the SNL feeder cells using CTK solution, broken down by pipetting, and seeded onto new MMC-treated SNL feeder cells in hiPSCs medium once a week. The 15M63 hiPSCs line was kindly provided from the Center for iPS Cell Research and Application of Kyoto University (Kyoto, Japan) and cultured on the vitronectin (Invitrogen, CA, USA)-coated dishes in Stem Fit(Ajinomoto, Tokyo, Japan). For passage, 15M63 was dissociated using accutase (Innovative Cell Technologies, San Diego, USA) by pipetting and inoculated on the vitronectin coated dishes and then cultured in Stem Fit supplemented with 10µM Y27632 (Cayman Chemical, Ann Arbor, MI, USA).

Differentiation of hiPSCs

Differentiation of hiPSCs was performed in suspension culture using bioreactors to form 3D spheroid. Undifferentiated 4M cells were detached from the feeder cells with CTK solution

and dissociated into single cells using Accumax (Innovative Cell Technologies). Then cells were seeded at a density of 1x10^6 cells/ml in a spinner type bioreactor (ABLE, Tokyo, Japan) containing 30ml of Essential 8 (Gibco) /StemFlex (Gibco) (1:1) with 10μM ROCK inhibitor. The next day, culture medium was exchanged for fresh Essential 8/StemFlex. One day later, culture medium was exchanged for hiPS medium, and differentiation was started the next day. Undifferentiated 15M63 cells were dissociated with Accutase and inoculated at a density of 1x10^6 cells/ml in the spinner type bioreactor containing 30ml of Stem Fit with 10μM ROCK inhibitor. The next day, culture medium was exchanged for fresh Stem Fit. One day later, culture medium was exchanged with hiPS medium and differentiation was started the same as 4M.

Stage1 (S1), Spheroids were cultured for 2 days in RPMI 1640 (FUJIFILM Wako) supplemented with 0.25% bovine serum albumin (BSA; Sigma, USA), 0.4x PS (FUJIFILM Wako), 1mM sodium pyruvate (FUJIFILM Wako), 1xNEAA, 80ng/mL recombinant human activin A (Peprotech), 55μM 2-ME, 50ng/ml FGF2, 20ng/ml recombinant bone morphogenetic protein 4 (BMP4; Peprotech) and 3μM CHIR99021 (Biovision, Milpitas, CA, USA). Then the spheroids were cultured for 1 day in RPMI 1640 supplemented with 1:200 Insulin, Transferrin, Selenium, Ethanolamine Solution (ITS-X; Gibco), 0.25% bovine serum albumin (BSA; Sigma), 0.4x penicillin and streptomycin, 1mM sodium pyruvate, 1x NEAA, 80ng/mL recombinant human activin A (Peprotech), and 55μM 2-ME. Next, the spheroids were cultured for 1 day in RPMI 1640 (FUJIFILM Wako) supplemented with 0.25% bovine serum albumin (BSA; Sigma), 0.4x PS, 1mM sodium pyruvate, 1x NEAA, 80ng/mL recombinant human activin A (Peprotech) and 55μM 2-ME.

S2, spheroids were cultured for 2 days in IMDM/Ham's F12 (3:1) supplemented with B27 (Gibco), N2 (Wako), 0.1% BSA, 0.4μM monothioglycerol (wako), 0.4x PS, 2.5μM Y27632, 1μM Dorsomorphin (wako) and 10μM SB431542 (wako). And then spheroids were cultured

for 1 days in IMDM/Ham's F12 (3:1) supplemented with B27 (Gibco), N2 (Wako), 0.1% BSA, 0.4μM monothioglycerol (wako), 0.4x PS, 2.5μM Y27632, 1μM IWP2 (wako) and 10μM SB431542. For 15M63, 0.25mM ascorbic acid (sigma) was also supplemented.

S3, spheroids were cultured for 9 days in IMDM/Ham's F12 (3:1) supplemented with B27 (Gibco), N2 (Wako), 0.1% BSA, 0.4μM monothioglycerol (wako), 0.4x PS, 3μM CHIR99021, 0.075μM RA (wako) and 10ng/ml BMP4 (Peprotech). For 15M63, 0.25mM ascorbic acid (sigma) was also supplemented. During S3, the medium was changed every other day.

S4, spheroids were cultured for 42-49 days in IMDM/Ham's F12 (3:1) supplemented with B27 (Gibco), N2 (Wako), 0.1% BSA, 0.4μM monothioglycerol (wako), 0.4x PS, 3μM CHIR99021, 0.050μM Dexamethasone (wako), 0.05 mM IBMX (Cayman), 0.025mM 8-bromo-cAMP (Selleck Chemicals, Huston, TX, USA), 10ng/ml FGF7 and 10ng/ml FGF10. For 15M63, 0.25mM ascorbic acid (sigma) was also supplemented. During S4, the medium was changed every other day.

Quantitative RT-qPCR for characterization of iPSC-derived spheroids

We extracted total RNA using RNAiso (Takara Bio, Shiga, Japan) from differentiated spheroids and cDNA was synthesized with PrimeScripit II reverse transcriptase (Takara Bio) using random nonamer and oligos (dT18). Quantitative RT-PCR reactions were run on the CFX96Touch Deep Well (Bio-Rad, Hercules, CA) using GoTaq qPCR master mix (Promega, Madison, WI). Relative quantification was performed against a standard curve, and the expression level of target genes was normalized against that of the reference gene, ornithine decarboxylase antizyme (OAZ1). The primer details of target or reference genes were as follows: AEC2; forward primer 5'-CAAGGTGACAGGTCTAAAGAGAGAAG-3', reverse primer 5'-GTTTCTATCAGGCATGCTCTGG-3', TMPRSS2; forward primer 5'-

GACTCCAAGACCAAGAACAATGAC-3’, reverse primer 5’-GAAATCCAGCAGAGCTGTTCTG-3’, VILLIN1; forward primer 5’-CAGGATGACTTGGAAGAGGATG-3’, reverse primer 5’-ATGGGTCTTGAGGTATTCCTGTG-3’, FULLIN; forward primer 5’-CTCGGTACACACAGATGAATGAC-3’, reverse primer 5’-CACCACAGACACCGTTGTTG-3’, CDX2; forward primer 5’-ACCGCAGAGCAAAGGAGAG-3’, reverse primer 5’-AGGCTTGCAGGGAAGACAC-3’, OAZ1; forward primer 5’-GTCAGAGGGATCACAATCTTTCAG-3’, reverse primer 5’-GTCTTGTCGTTGGACGTTAGTTC -3’.

Morphological and immunocytochemical characterization of iPSC-derived spheroids

Differentiated spheroids were fixed in 4% paraformaldehyde at RT for 30 min. After washing with PBS twice and with MilliQ water once, they were immersed in 70% ethanol, dehydrated in ethanol series and infiltrated with xylene. Then these cells were embedded in paraffin and cut into 3-μm sections. Hematoxylin and eosin staining was performed according to the standard protocol. Immunofluorescent staining was carried out with primary antibodies and fluorescein conjugated secondary antibodies. Primary antibodies included goat anti-ACE2, 1:400 (R&D, MN, USA. Cat # AF933), rabbit anti-TMPRSS2, 1:1000 (abcam, UK. Cat # ab92323), mouse anti-VILLIN1 1:50 (SantaCruz, NJ, USA. Cat # sc-58897). The following secondary antibodies were used: Alexa Fluor 488-conjugated donkey anti-goat IgG (Invitrogen. Cat # A-11055), Alexa Fluor 488-conjugated goat anti-rabbit IgG (Invitrogen. Cat # A-11008) and Alexa 594-conjugated goat anti-mouse IgG (Invitrogen. Cat # A-11005). Slides were then counterstained with 40, 60-diamidino-2-phenylindole (DAPI; Invitrogen) (0.14 μg/mL) prior to mounting with Fluoromount (Diagnostic Biosystems). Phase-contrast or fluorescent images were taken with a charge-

coupled device (CCD) camera (DP71; Olympus, Japan).

Preparation of differentiated cells for Single cell RNA sequence

Differentiated cells were washed with D-PBS(-) twice and added with 500μl of accutase. They were incubated at 37℃ for 20 min and dissociation was promoted by pipetting several times. Dissociated cells were rinsed with D-PBS(-) containing 0.1% BSA twice, and then dead cells were removed using Dead Cell Removal Kit (Miltenyibiotech, Bergish Gladbach, Germany) and LS columns (Miltenyibiotech). Collected cells were washed with D-PBS(-) containing 0.1% BSA and these cells were used for single cell RNA sequence.

RNA sequencing and sequencing data analysis

Single-cell library preparation was carried out with Chromium Next GEM Single Cell 5' Library & Gel Bead Kit v1.1 (10× Genomics, Pleasanton, CA). Cell suspensions were loaded onto a Chromium Single-Cell Chip along with the reverse transcription (RT) master mix and single-cell 5' gel beads, aiming for 5000 cells per channel. Following generation of single-cell gel bead-in-emulsions (GEMs), RT was performed using a BioRad T-100 (Bio-Rad Laboratories, Hercules, CA); 16 cycles were used for cDNA amplification. In order to construct the gene expression library, cDNA were processed according to the protocol for Chromium Single Cell 5' Library Construction Kit(10× Genomics) including 14 amplification cycles. To obtain TCR and BCR repertoire profile, VDJ enrichment for TCR and BCR were carried out with Chromium Single Cell V(D)J Enrichment Kit, Human T cell (10× Genomics) and Chromium Single Cell V(D)J Enrichment Kit, Human B Cell (10× Genomics), respectively. Each cDNA samples were used for VDJ enrichment with 9 cycles of amplification reaction and library construction according to the manufacture's protocol. Libraries were converted with MGIEasy Universal Library Conversion kit (MGI Tech Co.,

Ltd., Shenzhen, CHINA) and sequenced on an MGI DNBSEQ-G400 (MGI Tech Co., Ltd.) using a 150-pair-end sequencing kit. The 10× Genomics Cell Ranger pipeline (v5.0.0) was used to perform sample demultiplexing, alignment to the Hg38 reference genome, barcode/Unique Molecular Identifier (UMI) processing, and gene counting for each cell. The Seurat package (v. v4.0.1)(S1, S2) and R-4.0.5 were used for quality checking, filtering, normalization, clustering analyses, and visualization.

Cells, viruses, and antiviral compounds

VeroE6 cells and TMPRSS2-overexpressing VeroE6 ($VeroE6^{TMPRSS2}$) cells were obtained from American Type Culture Collectio (ATCC)(Manassas, VA, U.S.A) and the Japanese Collection of Research Bioresources (JCRB) Cell Bank (Osaka, Japan), respectively. VeroE6 cells were maintained in Dulbecco's modified Eagle's medium (d-MEM) supplemented with 10% fetal bovine serum (FCS), 100 μg/ml of penicillin, and 100 μg/ml of streptomycin. $VeroE6^{TMPRSS2}$ cells were maintained in d-MEM as mentioned above in the presence of 1 mg/ml of G418. SARS-CoV-2 strain JPN/TY/WK-521 ($SARS\text{-}CoV\text{-}2^{WK\text{-}521}$) was obtained from the National Institute of Infectious Diseases (Tokyo, Japan) and passaged 5 times on $VeroE6^{TMPRSS2}$ cells to obtain a working P5 stock. Titration of the P5 viral stock was performed on VeroE6 cells, with a limiting dilution technique, and the extracellular virus produced by infected VeroE6 cells was quantified with RT-qPCR assay. The quality control of $SARS\text{-}CoV\text{-}2^{WK\text{-}521}$ was performed with direct sequencing of the spike region and the biological activity assay using Vero E6 cells before the SARS-CoV-2 infection assays conducted in the present study. The antiviral agent Remdesivir was purchased (Selleck, Houston, TX). 5h was synthesized by A. K.Ghosh. The purity of each compound was >99% and the compounds were dissolved in DMSO at 20 mM as stock solutions.

Evaluation of SARS-CoV-2 replication capacity in the Spheroids.

Spheroids_$_{4M}^{ACE\text{-}TMPRSS2}$ and Spheroids_$_{15M63}^{ACE\text{-}TMPRSS}$, were exposed to SARS-CoV-2^{WK251} at multiplicity of infection (MOI) of 0.05. Three hours post exposure, the virus was washed out three times with culture medium, disseminated in 96-well plates (Corning® 96-well Clear Flat Bottom Ultra-Low Attachment Microplate) at the density of $2x10^5$ cells/well. The supernatants were then collected in the time course of days 0, 1, 2, and 3, and the amounts of virus in the supernatants were quantified by RT-qPCR. On day 3, spheroids were collected and the localization of SARS-CoV-2 infected cells and ACE2-positive cells were examined by immunocytostaining and TEM. General morphology of the Spheroids was observed with Phase-contrast microscopy over the incubation period. Details are shown in the following sections "**Antiviral activity and cytotoxicity assays**", "**Immunocytochemistry for SARS-CoV-2 infected cells**" and "**Transmission electron microscopy**" below.

Evaluation of infection inhibition effect by hACE2 blocking

To examine if the spheroids exhibit ACE2-mediated infection susceptibility to SARS-CoV-2 $^{WK\text{-}521}$, Spheroids_$_{4M}^{ACE\text{-}TMPRSS2}$ and Spheroids_$_{15M63}^{ACE\text{-}TMPRSS}$, were seeded into 96-well plates together with ten-fold serially diluted anti-hACE2 mAb (AC384) (Adiogen Corp. San Diego, CA, USA) (from40 μg/ml) or mouse IgG1 isotype control (15H6) (Adiogen Corp. San Diego, CA, USA) for 2hr. Then the Spheroids were collected and exposed to SARS-CoV-2^{WK251} at multiplicity of infection (MOI) of 0.05 in Eppendorf microtubes. Three hours post exposure, the virus was washed out three times with culture medium, disseminated in 96-well plates at the density of $2x10^5$ cells/well and incubated with the ten-fold serially diluted anti-hACE2 mAb or isotype control (from40 μg/ml) for 72 hours. On day3 post exposure, supernatants of culture medium were collected for quantitative analysis using RT-qPCR. Details are shown in the following section "**Antiviral activity and cytotoxicity**

assays” below.

Antiviral activity and cytotoxicity assays

Spheroids$_{_4M}^{ACE2\text{-}TMPRSS2}$ and Spheroids$_{_15M63}^{ACE2\text{-}TMPRSS2}$ were exposed with SARS-CoV-2 $^{WK\text{-}521}$ at 0.05 MOI in an Eppen tube, continuously incubated 3 hours, wash with culture medium 3 times, and then evenly plated on a 96-well cell culture plate at density~ $2x10^5$cell / well and incubated with or without test compounds for 72 hours. On day 3 post exposure, supernatants of culture medium and the Spheroids were collected. Viral RNA was extracted from the supernatants using a QIAamp viral RNA minikit (Qiagen, Hilden, Germany), and quantitative RT-PCR (RT-qPCR) was performed using One Step PrimeScript III RT-qPCR mix (TaKaRa Bio, Shiga, Japan) following the instructions of the manufacturers. The primers and probe used for detecting SARS-CoV-2 nucleocapsid (1) were 5’-AAATTTTGGGGACCAGGAAC-3’ (forward), 5’- TGGCAGCTGTGTAGGTCAAC-3’ (reverse), and 5’-FAM- ATGTCGCGCATTGGCATGGA-black hole quencher 1 (BHQ1)-3’ (probe). To determine the cytotoxicity of each compound, cells were seeded in a 96-well plate (2×10^5 cells/well). One day later, various concentrations of each compound were added, and cells were incubated for additional 3 days. The 50% cytotoxic concentrations (CC_{50}) values were determined using the WST-8 assay and Cell Counting Kit-8 (Dojindo, Kumamoto, Japan).

Immunocytochemistry for SARS-CoV-2 infected cells

Cultured spheroids were fixed with 4% paraformaldehyde–phosphate-buffered saline (PBS) for 15 min, washed with PBS three times for 5 min each time, and then blocked with a blocking buffer (10% goat serum, 1% bovine serum albumin [BSA], 0.3% Triton X-100, PBS 1x) for 1 h. After removal of the blocking buffer, the cells were immediately stained with a

convalescent IgG fraction, which was isolated from serum of a convalescent COVID-19 individual using a spin column-based antibody purification kit (Cosmo Bio, Tokyo, Japan) overnight at 4°C. The stained cells were washed with PBS (300 µl/well) three times for 5 min each time, and the cells were incubated with secondary antibody goat polyclonal anti-human IgG-Alexa Fluor 488 Fab fragment antibody (Jackson ImmunoResearch Laboratories, Inc., West Grove, PA, USA. Cat#109-547-003), together with Texas Red-X dye-conjugated phalloidin (Thermo Fisher Scientific, Cat#T7471) for F-actin visualization for 2 h. After washing of the cells with PBS (300 µl/well) three times for 5 min each time, DAPI (4',6-diamidino-2-phenylindole) solution, NucBlue™ Fixed Cell ReadyProbes™ Reagent, (Thermo Fisher Scientific, Cat#R37606) was further diluted with PBS to the final concentration of 0.14 µg/mL and 50 µl/well was added to stain nuclei. Signals were acquired with a Cytation 5 cell imaging multi-mode reader (BioTek, Winooski, VT, USA).

Transmission electron microscopy

Samples were pre-fixed with 2% paraformaldehyde and 2% glutaraldehyde (in 30 mM HEPES buffer containing 100mM NaCl and 2mM CaCl2) [pH 7.4]) overnight at 4°C, followed by post-fixation with an aldehyde–OsO4 mixture (1.25% glutaraldehyde, 1% paraformaldehyde, 0.32% K3[Fe(CN)6], and 1% OsO4 in 30 mM HEPES buffer [pH 7.4]) for 1 hours at room temperature. Fixed samples were washed three times with Milli-Q water (Merck Millipore Corporation, Tokyo, Japan), dehydrated in a graded ethanol series, infiltrated with propylene oxide, and embedded in Quetol 812 epoxy resin (Nisshin EM Corporation, Tokyo, Japan). Resin blocks were sectioned at 80 nm thickness with an ultramicrotome (Leica EM UC7, Leica), contrasted with uranyl acetate and lead citrate, and then observed with a transmission electron microscope (JEM-1400, JEOL Ltd., Tokyo, Japan).

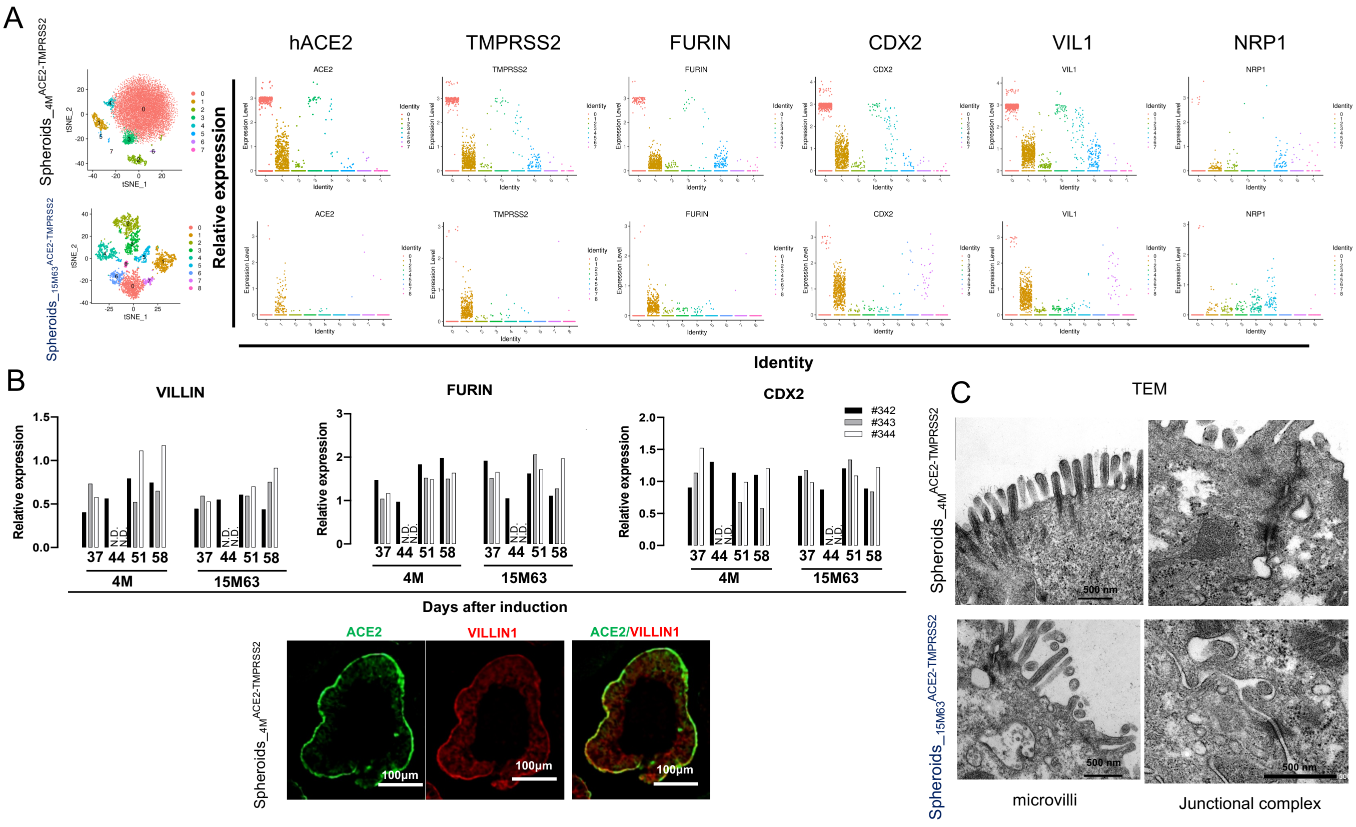

Supplementary Fig 1. Characterization of Spheroids$^{\text{ACE-TMPRSS2}}$.

(A) Differentiated cells in Spheroids$^{\text{ACE-TMPRSS2}}$ were examined with single-cell RNA sequence analysis, which revealed the properties of enterocytes. (B) The relative expression levels of enterocyte-related RNA to control (human small intestine) were examined with RT-qPCR, while the localization of the expressed proteins was visualized with immunocytostaining. #342, #343, and #344 indicate experiment-1, -2, and -3, respectively. Representative immunostaining images from three independently conducted experiments are shown. ACE2 antigens and VILLIN1 are indicated in green and red, respectively. (C) Microvilli and junctional complexes were observed with transmission electron microscopy. Representative electron microscopic images from three independently conducted experiments are shown. Details of the methods are described in the supplementary information.

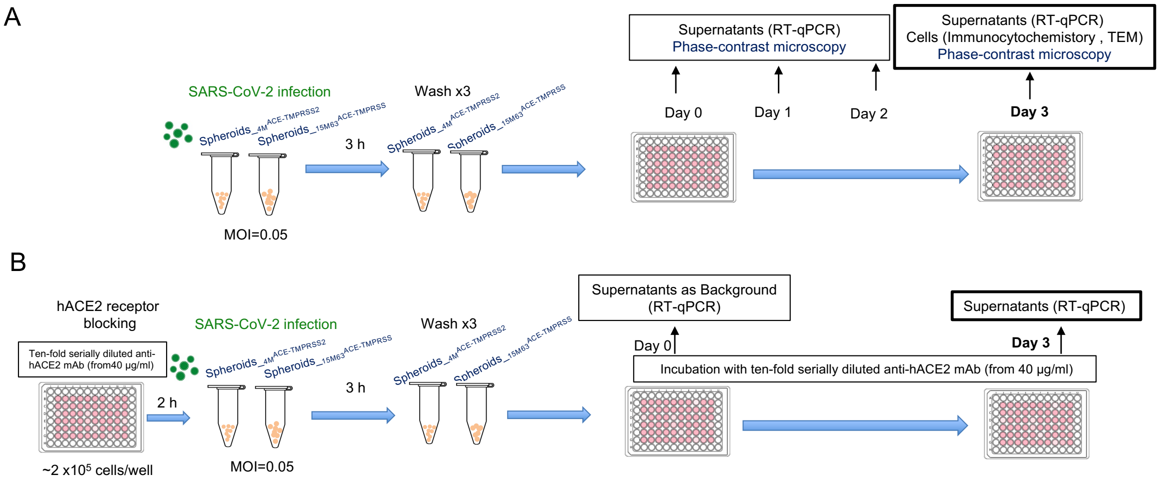

Supplementary Figure 2. Scheme of the experimental setups.

(A) Protocol for evaluation of SARS-CoV-2 replication capacity in the Spheroids. Spheroids_$_{4M}^{ACE\text{-}TMPRSS2}$ and Spheroids_$_{15M63}^{ACE\text{-}TMPRSS}$ were exposed to SARS-CoV-2^{WK251} at a multiplicity of infection (MOI) of 0.05. Three hours post-exposure, the virus was washed three times with culture medium, seeded in 96-well microtiter culture plates (clear flat-bottom ultra-low attachment microplate) at a density of $2x10^5$ cells/well. The supernatants were then collected in the time course of days 0, 1, 2, and 3, and the numbers of virus in the supernatants were determined with RT-qPCR. On day 3, spheroids were collected and the localization of SARS-CoV-2-infected cells and ACE2-positive cells were examined using immunocytostaining and TEM. General morphology of the Spheroids was obtained with phase-contrast microscopy over the incubation period. (B) Protocol for evaluation of infection inhibition effect by hACE2 blocking. Spheroids_$_{4M}^{ACE\text{-}TMPRSS2}$ and Spheroids_$_{15M63}^{ACE\text{-}TMPRSS}$ were seeded onto 96-well plates together with ten-fold serially-diluted anti-hACE2 mAb (AC384) (from 40 µg/ml) for 2 hr and then the Spheroids were collected and exposed to SARS-CoV-2^{WK251} at a multiplicity of infection (MOI) of 0.05 in Eppendorf microtubes. Three hours post-exposure, the virus was washed three times with culture medium, seeded in 96-well plates at a density of $2x10^5$ cells/well and incubated with the ten-fold serially-diluted anti-hACE2 mAb (from 40 µg/ml) for 72 hours. On day 3 post-exposure, the supernatants of the culture were collected for quantitative analysis using RT-qPCR.
